# Barnase-barstar Specific Interaction Regulates Car-T Cells Cytotoxic Activity toward Malignancy

**DOI:** 10.1134/S1607672922700041

**Published:** 2023-01-18

**Authors:** R. S. Kalinin, V. O. Shipunova, Y. P. Rubtsov, V. M. Ukrainskay, A. Schulga, E. V. Konovalova, D. V. Volkov, I. A. Yaroshevich, A. M. Moysenovich, A. A. Belogurov, G. B. Telegin, A. S. Chernov, M. A. Maschan, S. S. Terekhov, V. D. Knorre, E. Khurs, N. V. Gnuchev, A. G. Gabibov, S. M. Deyev

**Affiliations:** 1 Shemyakin-Ovchinnikov Institute of Bioorganic Chemistry, Russian Academy of Sciences, Moscow, Russia; 2 Dmitry Rogachev National Medical Research Center of Pediatric Hematology, Oncology, and Immunology, Moscow, Russia; 3 Moscow State University, Moscow, Russia; 4 Engelhardt Institute of Molecular Biology, Russian Academy of Sciences, Moscow, Russia; 5 National Research University Higher School of Economics, Moscow, Russia

**Keywords:** Barnase-barstar interaction, DARPins, CAR-T cells, solid tumors

## Abstract

The development of CAR-T specific therapy made a revolution in modern oncology. Despite the pronounced therapeutic effects, this novel approach displayed several crucial limitations caused by the complications in pharmacokinetics and pharmacodynamics controls. The presence of the several severe medical complications of CAR-T therapy initiated a set of attempts aimed to regulate their activity in vivo. We propose to apply the barnase-barstar system to control the cytotoxic antitumor activity of CAR-T cells. To menage the regulation targeting effect of the system we propose to use barstar-modified CAR-T cells together with barnase-based molecules. Barnase was fused with designed ankyrin repeat proteins (DARPins) specific to tumor antigens HER2 (human epidermal growth factor receptor 2) The application of the system demonstrates the pronounced regulatory effects of CAR-T targeting.

The development of anti-cancer therapeutics based on the genetically modified human blood cells displayed numerous positive results during the last decade [1, 2]. However, the lack of controlled pharmacokinetics and pharmacodynamics brings a substantial limitation in this approach showing a huge risk of the clinical side effects [3–6]. A numerous attempts to regulate tumor cytotoxicity caused by CAR-T cells were accomplished recently [7–9]. Some of them were concentrated on recruiting of FDA-approved drugs [10–14]. The specific protein-protein interaction may give a clue to solve the problem of an effective tuning of CAR-T cytotoxicity toward tumors. The RNase superfamily may be substantially inhibited by its cognate inhibitor barstar. The RNase-barstar complex is characterized by extremely tight binding with the extraordinary affinity (KD ∼10^–14^) [15, 16]. This makes sense to use the members of this complex for specific targeting of CAR-T cells and regulate cytotoxic antitumor effects. For this purpose, we designed two types of constructs, i.e. Barnase fused with previously designed ankyrin repeat proteins (DARPins) and barstar-modified CAR (BsCAR). We applied here the innovative approach for specific CAR-T targeting. We substituted the widely used targeting vectors based on the constructs from Ig superfamily by peptide scaffolds [17]. The applied DARPin is designed as a specific agent to tumor antigens HER2 (human epidermal growth factor receptor (Fig. 1).

**Fig. 1.**
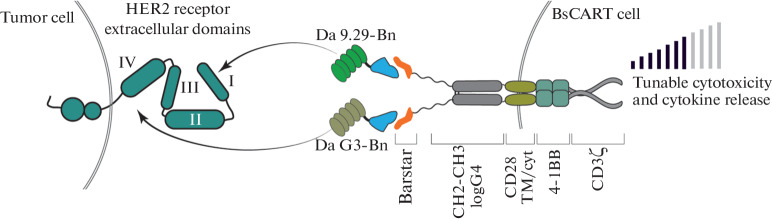
The scheme of regulating the specific targeting of a T-cell modified with a universal BsCAR by the protein darpin-barnase (Da-Bn) targeting the HER2 of the tumor cell. The HER2 receptor has 4 extracellular subdomains: membrane-proximal-IV specific Da G3-Bn, membrane-distal I Da 9.29-Bn. Barnase interacts with high affinity with its inhibitor-barstar, which acts as a recognition domain in the CAR construct. GGGSGGGSGGGS and CH2-CH3 IgG4 are used as a hinge, CD28 TM/cyt = transmembrane and cytoplasmic domains from CD28, 4-1BB = cytoplasmic activation domain from CD137, CD3ζ = cytoplasmic activation domain from CD3ζ.

We are showing here that the DARPin-barnase (DARPin-Bn) protein is allowing to regulate specific targeting of tumor cells by T cells modified with universal BsCAR. We prepared a fusion proteins of anti-HER2 DARPin 9.29 (18) was used here as molecular “vector” directing BsCAR T cells against HER2+ tumor cells. DARPins 9.29 can bind with different domains of the HER2 receptor interacts with the membrane-distal subdomain I, with KD = 0.09 nM [19] and KD = 3.8 nM [18] respectively. We designed the scheme of the in vivo experiments with mice (BALB/c nude mice) (Fig. 2).

**Fig. 2.**
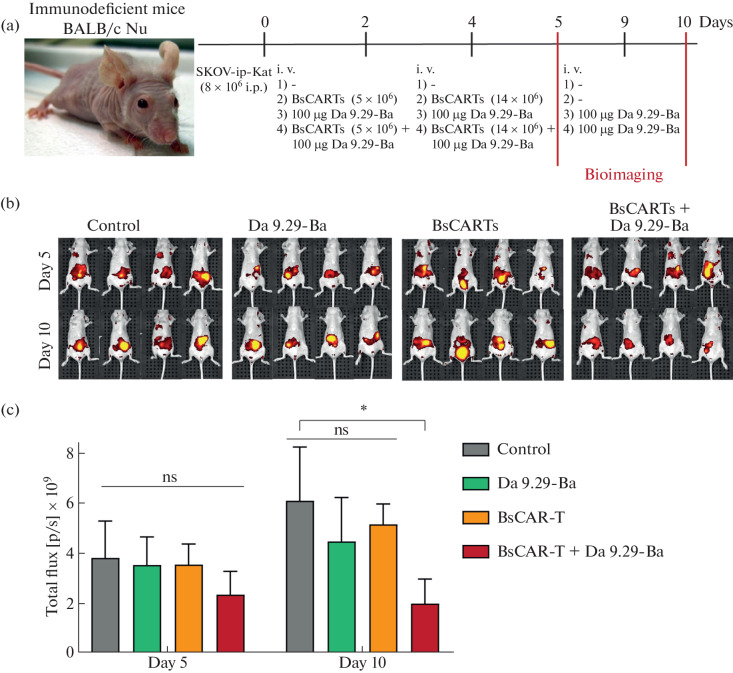
BsCAR T cells targeted by DARPin 9.29.-Bn suppress HER2-positive tumors in vivo. (a) Scheme of the experiment, where Balb/c Nu mice (females, 20–24 g) were intraperitoneally injected with 8 million SKOV-ip-Kat cells that form solid tumor nodes in the peritoneum. The presence of the red fluorescent protein TurboFP635 in the cytoplasm of these cells makes it possible to visualize the growth and spread of tumor nodes in mice using in vivo imaging technology using the IVIS SPectrum CT system (Perkin Elmer, USA). On day 2, 8 million, on day 4, 14 million BsCAR-T cells were intravenously injected into two groups of mice: the control group – BsCAR-T and the experimental group – BsCAR-T + darpin 9.29 – barnase. The therapy was performed by intraperitoneal administration of Da 9.29-Ba according to the scheme in the figure. Visualization of tumors was performed on the 5th and 10th days after the introduction of SKOV-ip-Kat cells. (b) IVIS images of animals treated with BsCAR-T cells alone or in combination with 9.29-barnase. The fluorescence of tumors of individual mice in each group is shown. (c) Tumor burden quantified as the mean fluorescence over 5 and 10 days. Data were analyzed using the Mann-Whitney test and presented as mean ± range. Statistical significance: *p < 0.05.

We may see from the data displayed on figure 1 that the proposed Barnase-Barstar system may specifically regulate antitumor cytotoxic effect of CAR T therapy. This system may control the efficacy of this therapy and may prevent the complications of CAR-T therapy and side effects.

Here we are showing the Modular CAR approach which is based on universal CARs that allows to separate two distinct process: (1) antigen recognition and (2) CAR T activation. This process might have perspectives shoving different redirecting agents. This may give rise the platform for CAR-T directed toward solid tumors.

## METHODS

### Obtaining BsCAR-T Cells

Obtaining a BsCAR lentiviral construct, producing CAR-T cells, and determining the level of BsCAR expression have been described previously [20]. The experiments used BsCAR-T cell products with a transduction level of 50% in the first cycle of administration and 40% in the second cycle of administration of BsCAR-T cells intravenously to immunodeficient BALB/c Nu mice.

### In vivo Study

BALB/c Nu female mice, 8 weeks old, weighing 20–24 g, were taken for experiments. Animals were housed under specific pathogen-free conditions in the Pushchino Animal Breeding Facility IBCh RAS (Bioresource collection “Collection of laboratory rodents SPF category for basic, biomedical and pharmacological research”). Mice were intraperitoneally inoculated with 8 × 10^6^ SKOV-ip-Kat cells, developed earlier [21], in 60% Matrigel in 100 µl of complete culture medium. On the second day, mice were randomized into 4 groups (*n* = 4). Animals from the 2nd and 4th groups were injected intravenously with 5 × 10^6^ million on the 2nd day and 14 × 10^6^ million on the 4th day of BsCAR-T cells. Four hours after injection of BsCAR-T cells, mice were intravenously injected intravenous of Da 9.29-barnase 100 μg in 50 μl PBS. The injection of Da 9.29-barnase was repeated on the 9th day of the experiment. The mice were weighed every other day and using the IVIS Spectrum In vivo Imaging System (PerkinElmer) on days 5 and 10.

### Statistical Analysis

Statistical processing of the experimental results was performed using the Prism 9 software package (GraphPad Software). Tumor fluorescence value measurements were statistically processed using one-way analysis of variance (ANOVA) and analyzed using the Mann-Whitney test. Values are presented as mean ± range (*n* = 4). Statistical significance: *p < 0.05.
